# Hepatitis C Virus; Its Implication for Endodontists

**Published:** 2014-07-05

**Authors:** Nima Mahboobi, Nastaran Mahboobi, Parvin Oliaei, Seyed Moayed Alavian

**Affiliations:** a Department of Endodontics, Tehran University of Medical Sciences, Tehran, Iran; b Marien Hospital Euskirchen, Lehrkrankenhaus der Uni, Bonn, Germany; c International Branch of Shiraz University of Medical Sciences, Shiraz, Iran; d Middle East Liver Diseases Center (MELD center), Tehran, Iran

**Keywords:** Blood-Borne Infections, Dentist, Diagnosis, Endodontics, Health Care Workers, HCV, *Hepatitis C Virus*, Needle-stick

## Abstract

Over 170 million patients worldwide are chronically infected with *Hepatitis C virus* (*HCV*); making it a globally important infection. Dentists constantly handle sharp instruments infected with biological fluids and are therefore considered at high-risk of contracting *HCV* infection. Needle-stick injuries seem to be the most common route of exposure to blood-borne pathogens in dental practice. Moreover, endodontist’s constant use of sharp instruments such as endodontic files with limited operative vision in a small working field (*i.e.* root canal system) increases their risk of exposure to infection. The aim of this study was to review the epidemiology of *HCV* infection in dental healthcare staff and the tests required for its diagnosis. We also look at the protocols for dental treatment in infected individuals and screening and dental examination tailored for *HCV* patients.

## Introduction

*Hepatitis C virus (HCV)*, also known as parentally transmitted or post transfusion non-A non-B hepatitis, was first identified in 1989. It is a single-stranded positive-sense RNA virus, enclosed in an envelope with a diameter of ~50 nm and is classified in a separate genus of *Hepacivirus* as the third member of the *Flaviviridae* family (the other two genera remain as *Pestivirus* and *Flavivirus*) [1, 2].

With more than 3% of world’s population being chronically infected (170 million individuals) in two decades, *HCV* infection is now considered a globally significant disease [2, 3]. The prevalence of *HCV* infection is estimated to be higher than *human immunodeficiency virus *(*HIV*) or even *hepatitis B virus *(*HBV*), in the USA. Infection with *HCV* is the most leading cause of liver transplantation worldwide. The most important route of transmission before 1989 was blood transfusion [4-6]. Mortality rate of *HCV* chronic infection is increasing in many parts of the world [5]. The prevalence of *HCV* infection is estimated to be 0.16% in Iran [7].

Six major genotypes (namely 1 to 6) of *HCV* with different geographically distribution have been identified so far. Genotypes 1, 2 and 3 are distributed worldwide with genotype 1 accounting for 40-80% of all infected cases. Genotype 4 is generally found in the Middle East and Egypt, genotype 5 in South Africa and genotype 6 in South-East Asia [1, 8].

To date no *HCV*-effective vaccine has been developed. In addition, treatments are focused upon achieving a sustained virological response with a combination of interferon and ribavirin, which are usually accompanied with some side effects [9-11]. 

The routes of disease transmission include intravenous drug abuse, non-protected sexual contact with multiple partners, iatrogenic acquisition (*e.g.* haemodialysis), accidental exposure such as needle-stick injuries (NSI) and vertical transmission from mother to the infant. However, in 30-40% cases of *HCV* infection, the route of acquisition cannot be identified [12, 13]. The results of a recently published paper showed that although weak, there is an undoubted risk of *HCV* transmission and cross contamination in dental care environment, and during dental treatments especially when precautionary methods fail [14].

In recent years, more attention has been dedicated to educate undergraduate and postgraduate dental students to enhance their knowledge on prevention of blood-borne infections (BBI). The results of recent studies on dental students’ knowledge on blood-borne pathogens show that the level of students’ knowledge about infectious diseases and their routes of transmission, increases as they enter higher academic semesters [20, 21]. Moreover, senior students showed a more positive approach and less discriminating attitudes toward patients with BBI in comparison with junior students. It seems that using these programs prepares the future dentists for their up-coming challenges when managing patients with BBI. In addition, periodic mandatory attendance of dental health care workers (DHCW) in continuing education programs will keep them up-to-date regarding this issue.

**Table 1:** The results of the recent studies on the prevalence of *HCV* infection among dental health care workers (DHCWs)

**Country**	**Population under study**	**Test**	**N exposed(% )**	**Main conclusion**
**Brazil, 2009 [**15**]**	Dentists registered at the Minas Gerais Dental Council and working regularly in Belo Horizonte	Serum anti-*HCV*	1302 (0.9%)	The seroprevalence of anti-*HCV* among dentists was low. No occupational exposure conditions were associated to the seroprevalence of *HCV*.
**Brazil, 2006 [**16**]**	Dentists working in a town in the state of Sao Paulo in Brazil	Serum anti-*HCV*; confirmed by PCR	135 (0.7%)	The study alerts using standard precautions during professional dental practice to avoid occupational acquisition of *HBV* and *HCV*.
**Israel, 2009 [**17**]**	Dentists attending an annual dental conference	Serum anti-*HCV*	296 (0.33%)	The study did not mention *HCV* as a hazard to dental professionals. However, infection control guidelines should be strictly followed.
**Japan, 2008 [**18**]**	42 dentists, 35 dental hygienists, 41 dental assistants, 8 dental mechanics and 15 clerks	Serum anti-*HCV*	141 (0%)	Being a DHCW is not associated with *HCV* infection.
**Germany, 2000 [**19**]**	215 dentists and 108 dental assistants attending the 1997 annual meeting of the Berliner Zahnärztekammer	Serum anti-*HCV*	323 (0.3%)	These results suggest that occupational transmission of *HCV* in dental settings occurs sometimes, but not frequently. Infection with *HBV* is 25 times higher.

Although different aspects of *HIV* infection has been studied in dentistry, there is rather low information regarding *HCV* infection in dental practice [22]. Most of the reports have been performed on different groups of DHCWs with variable subjects and methodology. Therefore, the aim of this study is to review the implications of *HCV* in endodontic practice; including the precautionary methods and universal cross-infection control protocols during daily practice as well as post-exposure strategies.

***HCV infection in DHCWs***

Dentists can be exposed to biological fluids through skin and mucous membranes or through percutaneous injuries, with the latter being the most common route of exposure. Blood exposure incidents, which may cause the transmission of blood-borne viruses, occur regularly during dental treatment procedures due to the close proximity to patient tissues, sharp instruments such as endodontic files, limited visual field in a small working area as well as the frequent patient movements during endodontic practice [23-25]. The risk of being infected with *HCV* after a single needle-stick injury is 3% [15, 20]. Using the anesthetic syringe and incorrect needle recapping procedures holding the cap with one hand and re-sheathing the needle with the other, are shown to be the most important causes of NSI in dentists and dental hygienists [26]. The risk of *HCV* infection following a blood splash is unknown but is believed to be greater if the source patient is positive for *HCV*-RNA and does not occur if patient is *HCV*-RNA negative [27].

The results of a nationwide study in the Netherlands showed that blood exposure incidents occur frequently in dental settings, irrespective of the type of practice. Because of such accidents, dental clinics need to have a protocol for handling blood exposure incidents according to safe working practice guidelines [23]. There are few reports on epidemiology of *HCV* infection in DHCWs [16, 17, 19]. According to our search results, no previous report has been performed particularly on endodontists. Although the World Health Organization (WHO) states that dentists are at greater risk of HCV infection, there are studies showing that the prevalence of *HCV* infection in this group is similar or even lower than that of the general population [15-19]. The prevalence of *HCV* infection in DHCWs is shown in Table 1.

**Table 2:** *HCV* diagnostic tests

**Anti-** ***HCV***	***HCV*** **-RNA**	**Interpretation**
**Positive **	Positive	Acute or chronic infection
**Positive**	Negative	Treated infection; below detectable levels of *HCV*-PCR test or anti-*HCV* false positive (less than 1%)
**Negative**	Positive	Early infection or chronic infection in immunosuppressed individuals
**Negative**	Negative	Non-infected

Previous papers demonstrated that the possibility of acquiring *HCV* infection is commonly related to age and work experience [16]. Also, a study performed in Brazil showed that anti-*HCV* seropositivity in DHCWs is significantly associated with a history of blood transfusion and a serologic test of *HCV* [15].

According to the results of the available reports, *HCV* infection in dentists is similar or lower than that of the general population. However, due to the importance of probable transmission of* HCV* in dental practice, the issue is more significant. Using standard precautionary methods prevent both dentists and their patients from being infected.

***Serologic tests***

Most *HCV*-infected patients (60 to 70%) are asymptomatic. When symptoms do occur, they are nonspecific including fatigue, nausea, anorexia, myalgia, arthralgia, weakness, and weight loss [28]. It has been demonstrated that patients’ medical histories are unreliable in identifying the exposure to blood-borne pathogens [29]. Dentists should be aware of *HCV* diagnostic tests which patients might be referred with. Although corresponding data shows that body fluids such as saliva and urine contain viral particles, routine diagnostic tests are still performed on serum samples [5, 30].

*HCV* diagnostic tests include the *HCV* antibody enzyme immunoassay (EIA), different generations of recombinant immunoblot assay (RIBA-1, 2 or 3) and quantitative *HCV* RNA-polymerase chain reaction (PCR). The most widely used initial assay for detecting *HCV* antibodies is the EIA. Quantitative viral load tests measure the amount of virus in blood. Quantitative studies provide information on initial viral load, viral load reduction with therapy and a sustained virologic response, defined as undetectable *HCV* by PCR, six months after ceasing the therapy. However, levels of* HCV*-RNA do not correlate directly with liver injury, duration of infection or disease severity. Table 2 explains the results and interpretation of* HCV* diagnostic tests. To analyze the liver function, patients might undergo some blood tests for assessment of liver enzymes as well as liver biopsy [28, 31-33].

Detection and quantification of *HCV*-RNA is useful in order to*: i)* diagnose a chronic *HCV* infection; *ii)* identify patients who need antiviral therapy; *iii)* monitor the virological responses to antiviral therapy; and *iv)* identify amino acid substitutions responsible for resistance to specific inhibitors of *HCV* viral proteins [33].

Recently the application of oral fluid for the detection and diagnosis of *HCV* infection has been evaluated repeatedly. Although most studies demonstrated that oral fluids [saliva and more specifically the gingival crevicular fluid (GCF)] can be used in *HCV* diagnosis, the appropriate measuring tool with reliable specificity is not available yet. More studies are recommended to design appropriate tests and validate tools [5].

***Dental treatment for HCV-infected patients***

Living with *HCV*, comes along with an array of potentially serious consequences. Apart from physical/medical issues, social stigma is also an issue that viral hepatitis patients might face [22].

National and international guidelines such as the needle-stick safety act in 2001, were developed to help minimizing the risk of exposure to blood-borne pathogens in DHCWs including dental personnel [26]. While dentists and non-infected patients can be infected with hepatitis following inadequate sterilization and other precautionary methods, adequate measures should definitely be taken [34].

One of the most significant phenomena associated with hepatitis infected patients is DHCWs’ fear of treating them. *HCV*-infected patients have significant oral health needs [35]. Unfortunately, the results of previous studies showed that a number of DHCWs prefer not to treat patients affected with *HCV*. Such a behavior is not only unethical but also induces patients to conceal parts of their medical history which will be detrimental for both patient and health care worker.

The results of a study aiming at evaluating DHCWs attitudes toward caring for people with *HCV* performed in Japan showed that significant associations exist among DHCW’s knowledge about *HCV* and their self-reported behavior towards this population. Thirty percent of dentists indicated that patients with *HCV* should be given the last appointment of the day. Dentists, who reported that they complied with infection control guidelines, were significantly more likely to treat people with *HCV*. Additionally, 14% of dentists stated that they did not want to treat injecting drug users [36]. The results of a study performed in Australia showed that all dentists perform dental treatment on the patients with *HCV* even without changing their personal protective methods [22].

Dentists should keep in mind that the best patients are those who inform us about their blood contamination. If we want them to inform us of the issue, our behavior should be non-discriminative and understanding. Our practice should be the same for all patients as they may carry a blood-borne virus unknown to us and/or themselves.

***Dental check-ups for HCV infected patients***

Previous reports showed that infection with *HCV* might accompany a number of extrahepatic conditions. Mixed cryoglobulinemia, glomerulonephritis, polyarteritis nodosa, rashes, renal disease, neuropathy and lymphoma are strongly associated with *HCV* infection. In addition, some oral conditions have been found to be related to *HCV* [37, 38].

There are just few reports on oral health of HCV infected patients, mostly performed in developed countries. The results of all these investigations show serious oral health needs in this group of patients [39, 40]. There are also oral mucosa conditions related to *HCV* infection. Reports show that oral lichen planus (OLP) is also associated with virus infection in some parts of the world [41, 42]. Replication of viral particles in salivary glands as well as antiviral therapies might cause symptoms similar to Sjogren’s syndrome and hyposalivation [43]. Besides, other conditions related to *HCV* infection such as oral cancer and pemphigus need to be assessed more precisely.

Dental problems might delay the administration of *HCV* treatments. Plus, dental problems might worsen after applying antiviral treatments because of their side effects [44]. Patients with *HCV* infection should be routinely screened for oral health problems. Dentists should also be aware of probable and common conditions in the oral mucosa in this population and should meticulously assess their patients.

## Conclusion

*HCV* infection is a condition with global impact. Along with common routes of transmission, DHCWs are always suspected whether to infect their patients or get infected. Although there are reports informing that dentists and oral surgeons have infected their patients with *HBV* during dental treatments, to date there is no similar report on *HCV*. However, available reports imply such threat in dentistry if universal infection control strategies are not adhered to. Further studies are also needed to assess the prevalence of *HCV* among DHCWs.

## References

[1] Pol S, Vallet-Pichard A, Corouge M, Mallet VO (2012). Hepatitis C: epidemiology, diagnosis, natural history and therapy. Contrib Nephrol.

[2] Lavanchy D (2009). The global burden of hepatitis C. Liver Int.

[3] El-Serag HB (2012). Epidemiology of viral hepatitis and hepatocellular carcinoma. Gastroenterology.

[4] Foster GR (2009). Recent advances in viral hepatitis. Clin Med.

[5] Mahboobi N, Porter SR, Karayiannis P, Alavian SM (2012). Oral fluid and hepatitis A, B and C: a literature review. J Oral Pathol Med.

[6] Alavian SM, Ahmadzad-Asl M, Lankarani KB, Shahbabaie MA, Bahrami Ahmadi A, Kabir A (2009). Hepatitis C infection in the general population of Iran: a systematic review. Hepat Mon.

[7] Zidan A, Scheuerlein H, Schüle S, Settmacher U, Rauchfuss F (2012). Epidemiological pattern of hepatitis B and hepatitis C as etiological agents for hepatocellular carcinoma in Iran and worldwide. Hepatitis monthly.

[8] Modi AA, Liang TJ (2008). Hepatitis C: a clinical review. Oral Dis.

[9] Schaefer EA, Chung RT (2012). Anti-hepatitis C virus drugs in development. Gastroenterology.

[10] Jun DW, Tak WY, Bae SH, Lee YJ (2012). Recent trends in the treatment of chronic hepatitis C. Korean J Hepatol.

[11] Casey LC, Lee WM (2012). Hepatitis C therapy update. Curr Opin Gastroenterol.

[12] Januszkiewicz-Lewandowska D, Wysocki J, Rembowska J, Pernak M, Lewandowski K, Nowak T, Nowicka-Kujawska K, Nowak J (2003). Transmission of HCV infection among long-term hospitalized onco-haematological patients. J Hosp Infect.

[13] Alter MJ (2011). HCV routes of transmission: what goes around comes around. Semin Liver Dis.

[14] Mahboobi N, Porter SR, Karayiannis P, Alavian SM (2013). Dental Treatment as a Risk Factor for Hepatitis B and C Viral Infection A Review of the Recent Literature. J Gastrointestin Liver Dis.

[15] Resende VL, Abreu MH, Paiva SM, Teixeira R, Pordeus SA (2009). Factors associated with seroprevalence of hepatitis C among dentists at a large Brazilian city. Virol J.

[16] Bellíssimo-Rodrigues WT, Machado AA, Bellíssimo-Rodrigues F, Nascimento MP, Figueiredo JFC (2006). Prevalence of hepatitis B and C among Brazilian dentists. Prevalence.

[17] Ashkenazi M, Fisher N, Levin L, Littner MM (2009). Seroepidemiology of hepatitis C antibodies among dentists and their self-reported use of infection control measures. Community Dent Health.

[18] Nagao Y, Matsuoka H, Kawaguchi T, Ide T, Sata M (2008). HBV and HCV infection in Japanese dental care workers. Int J Mol Med.

[19] Ammon A, Reichart PA, Pauli G, Petersen LR (2000). Hepatitis B and C among Berlin dental personnel: incidence, risk factors, and effectiveness of barrier prevention measures. Epidemiol Infect.

[20] Brailo V, Pelivan I, Skaricic J, Vuletic M, Dulcic N, Cerjan-Letica G (2011). Treating patients with HIV and Hepatitis B and C infections: Croatian dental students' knowledge, attitudes, and risk perceptions. J Dent Educ.

[21] Alavian SM, Mahboobi N, Savadrudbari MM, Azar PS, Daneshvar S (2011). Iranian dental students' knowledge of hepatitis B virus infection and its control practices. J Dent Educ.

[22] Temple-Smith M, Jenkinson K, Lavery J, Gifford SM, Morgan M (2006). Discrimination or discretion? Exploring dentists' views on treating patients with hepatitis C. Aust Dent J.

[23] van Wijk PT, Meiberg AE, Bruers JJ, Groenewold MH, van Raalten AL, Dam BA, Schneeberger PM (2012). The risk of blood exposure incidents in dental practices in the Netherlands. Community Dent Oral Epidemiol.

[24] Zarra T, Lambrianidis T (2013). Percutaneous injuries amongst Greek endodontists: a national questionnaire survey. Int Endod J.

[25] Mahboobi N, Agha-Hosseini F, Safari S, Lavanchy D, Alavian SM (2010). Hepatitis B virus infection in dentistry: a forgotten topic. J Viral Hepat.

[26] Shah SM, Merchant AT, Dosman JA (2006). Percutaneous injuries among dental professionals in Washington State. BMC Public Health.

[27] Gupta N, Tak J (2011). Needlesticks injuries in dentistry. Kathmandu Univ Med J (KUMJ).

[28] Wilkins T, Malcolm JK, Raina D, Schade RR (2010). Hepatitis C: diagnosis and treatment. Am Fam Physician.

[29] Ghadir MR, Belbasi M, Heidari A, Jandagh M, Ahmadi I, Habibinejad H, Kabiri A, Ghanooni AH, Iranikhah A, Alavian SM (2012). Distribution and risk factors of hepatitis B virus infection in the general population of Central Iran. Hepat Mon.

[30] Caldeira PC, Oliveira e Silva KR, Silva TA, Mattos Camargo Grossmann S, Teixeira R, Carmo MAVd (2012). Correlation between salivary anti-HCV antibodies and HCV RNA in saliva and salivary glands of patients with chronic hepatitis C. J Oral Pathol Med.

[31] Albeldawi M, Ruiz-Rodriguez E, Carey WD (2010). Hepatitis C virus: Prevention, screening, and interpretation of assays. Cleve Clin J Med.

[32] Chevaliez S, Pawlotsky JM (2009). Virological techniques for the diagnosis and monitoring of hepatitis B and C. Ann Hepatol.

[33] Chevaliez S, Pawlotsky JM (2008). Diagnosis and management of chronic viral hepatitis: antigens, antibodies and viral genomes. Best Pract Res Clin Gastroenterol.

[34] Ilguy D, Ilguy M, Dincer S, Bayirli G (2006). Prevalence of the patients with history of hepatitis in a dental facility. Med Oral Patol Oral Cir Bucal.

[35] Henderson L, Muir M, Mills PR, Spence E, Fox R, McCruden EA, Bagg J (2001). Oral health of patients with hepatitis C virus infection: a pilot study. Oral Dis.

[36] Richmond JA, Dunning TL, Desmond PV (2007). Health professionals' attitudes toward caring for people with hepatitis C. J Viral Hepat.

[37] Ali A, Zein NN (2005). Hepatitis C infection: a systemic disease with extrahepatic manifestations. Cleve Clin J Med.

[38] Mayo MJ (2003). Extrahepatic manifestations of hepatitis C infection. Am J Med Sci.

[39] Coates EA, Brennan D, Logan RM, Goss AN, Scopacasa B, Spencer AJ, Gorkic E (2000). Hepatitis C infection and associated oral health problems. Aust Dent J.

[40] Griffin SO, Barker LK, Griffin PM, Cleveland JL, Kohn W (2009). Oral health needs among adults in the United States with chronic diseases. J Am Dent Assoc.

[41] Lodi G, Pellicano R, Carrozzo M (2010). Hepatitis C virus infection and lichen planus: a systematic review with meta-analysis. Oral Dis.

[42] Mahboobi N, Agha-Hosseini F, Lankarani KB (2010). Hepatitis C virus and lichen planus: the real association. Hepat Mon.

[43] Aghemo A, Rumi MG, Monico S, Banderali M, Russo A, Ottaviani F, Vigano M, D'Ambrosio R, Colombo M (2011). Ribavirin Impairs Salivary gland function During Combination Treatment With Pegylated Interferon Alfa-2a In HEpatitis C patients. Hepat Mon.

[44] Nagao Y, Sata M (2010). Dental problems delaying the initiation of interferon therapy for HCV-infected patients. Virol J.

